# Enhancing Bandwidth and Efficiency with Slotted Ground Planes Embedding Antenna Boosters

**DOI:** 10.3390/mi16030250

**Published:** 2025-02-23

**Authors:** Sabrina Arús, Joan Navarro, Joan L. Pijoan, Aurora Andújar, Jaume Anguera

**Affiliations:** 1 Smart Society Research Group, Universitat Ramon Llull, 08022 Barcelona, Spain; sabrina.arus@salle.url.edu (S.A.); joanlluis.pijoan@salle.url.edu (J.L.P.); 2 Ignion, 08017 Barcelona, Spain; aurora.andujar@ignion.io

**Keywords:** antenna booster, antenna efficiency bandwidth potential, IoT, matching network, slotted ground plane, wireless device

## Abstract

The deployment of wireless devices has increased exponentially in recent years, not only for mobile applications but also for IoT. Typically, these IoT devices exchange data with other devices by means of wireless connections, where battery consumption depends on the antenna system’s efficiency. In applications where long battery life and reliable transmission are essential, improving the efficiency of the antenna is crucial. This study aims to investigate how shaping the ground plane of a wireless device can enhance bandwidth and antenna efficiency, specifically in low-frequency bands of 824–960 MHz, a common frequency band used in IoT where transmitting a small amount of data provides long battery life. Specifically, this work shows that by adding a slot in the ground plane, the current distribution is enlarged, which enables the excitation of its fundamental mode and, consequently, enhances the bandwidth and antenna efficiency by 2 dB. This approach is assessed using three different printed circuit boards (PCBs) that aim to characterise different form factors of IoT devices. A physical prototype is built to validate the results obtained in simulations.

## 1. Introduction

Recently, two main challenges have emerged in the field of antenna design for wireless devices: (1) accommodating antennas within small ground planes [1] and (2) achieving high efficiency at lower frequencies despite the complex geometries required [2]. As technology advances, devices are becoming smaller and more compact [3,4]. These reduced physical dimensions limit the antenna’s performance, especially at lower frequencies where larger structures are typically required for efficient radiation [5,6]. This situation is particularly concerning for small wireless devices such as small IoT trackers, where the limited physical space available for the antenna system forces the use of intricate designs [7], which typically reduce antenna efficiency.

In addition, IoT devices need to exchange data, and this communication requires efficient energy management to avoid excessive impact on battery consumption [8]. Antenna efficiency plays a critical role in this context. If the antenna operates at low efficiency, it will require more power to maintain reliable data transmission, directly impacting the device’s power consumption. This is particularly critical for devices designed to work in remote environments. Improving antenna efficiency is essential in these cases to minimise power consumption while ensuring consistent data exchange.

A common approach to address these challenges and mitigate these issues is the use of antenna booster technology [9], which relies on the use of a tiny component called an antenna booster. These non-resonant electrically small components (~λ/20, ~λ/30) rely on the ground plane to radiate [10]. The design complexity is then shifted to a matching network (MN) that adjusts the operating frequency [9]. However, when the size of the ground plane remains small relative to the wavelength (L < λ/π), it is challenging to obtain bandwidth and efficiency, which is not only a limitation of antenna booster technology but of antennas in general.

In order to overcome the limitation of small ground planes, this paper combines an antenna booster with slotted ground planes to enhance bandwidth and efficiency. To achieve this, slotted ground planes are proposed to improve the system’s bandwidth and efficiency.

Several studies have investigated the use of slots in ground planes to improve antenna performance in mobile devices. For example, the design of PIFA antennas with slotted configurations increases the bandwidth over different ground plane sizes, as observed in [11,12], where multiband enhancement is achieved in a 90 mm × 40 mm ground plane and a 107 mm × 40 mm configuration at 900 MHz, respectively. Similarly, the use of an antenna booster, such as the 5 mm × 5 mm × 5 mm in [13], improves the efficiency on a 100 mm × 40 mm ground plane, enhancing performance at multiband frequencies. Other approaches, as presented in [14], show that the use of slots in the ground plane for multilayer antennas is also effective in increasing the bandwidth of multiband PIFA antennas. In addition, other work reduces antenna height while maintaining a stable bandwidth [15]. These studies support the idea that slotted ground plane designs improve multiband bandwidth performance. However, they remain limited to the operating frequency determined by the antenna geometry. In [13], this limitation is addressed as the booster is a non-resonant element and, therefore, only depends on the size of the ground plane and matching network to resonate. However, like the other studies, it focuses on dimensions for mobile devices (~0.3λ at 900 MHz). This paper aims to provide a solution for smaller platforms (~0.15λ at 900 MHz) (Table 1) and to analyse new aspects, such as the optimal slot position and how lowering the quality factor (Q) of the ground plane reduces the matching network losses.

In this regard, the antenna booster, with the addition of the matching network, is responsible for controlling the operating frequency. At the same time, the slot acts as a performance enhancement mechanism by modifying the current distribution on the ground plane. Assigning the frequency control to the antenna booster enables improved performance even at lower frequencies, such as those within cellular bands (824 MHz–960 MHz).

As a result, this article’s main contribution is a demonstration of how the use of electrically small (length ~0.15λ) slotted ground planes embedding antenna boosters can effectively increase the total efficiency of wireless antenna systems.

This paper is structured as follows: Section 2 reviews the concepts necessary to understand the paper; Section 3 discusses the experiments conducted for the study, including the simulation of the reference PCB, the simulation of the slotted ground plane; Section 4 presents the implementation and comparison of the solution, as well as a practical case involving battery performance. Finally, Section 5 presents the conclusions.

## 2. Background

### 2.1. Antenna Booster

In conventional antenna design, antennas are typically tuned to resonate at specific frequencies based on electromagnetic theory [16]. In contrast, an antenna booster works differently as it does not resonate at a specific frequency. Instead, it relies on the ground plane to facilitate the radiation process [9]. The non-resonant booster acts as a catalyst, exciting multiple radiation modes through the ground plane.

The design of the antenna booster represents a significant advance in achieving effective radiation within compact systems. As a non-resonant element, its performance is dependent on the dimensions and configuration of the ground plane. Achieving wide bandwidth and high efficiency is a significant challenge, not only for antenna boosters but for any other antenna technology, when working with small ground planes (length < λ/π) [9] due to the limited space available to support the current distributions required for effective radiation.

Strategic placement of the antenna booster on the PCB is key for efficient excitation of the ground plane radiation modes. The antenna booster is typically placed in a corner of the ground plane where it properly excites the desired current distributions [9]. Other antenna boosters (magnetic) work at the central edge of the ground plane. This configuration enhances the interaction between the antenna booster and the ground plane, allowing the excitation of the fundamental mode [17], which is essential for efficient radiation, particularly in the low-frequency bands 824–960 MHz analysed in this study.

### 2.2. Antenna Parameters

The total efficiency of an antenna (*η_t_*), defined as the ratio of the radiated power to space (*P_r_*) over the power delivered by the transceiver (*P_i_*) is influenced by several antenna parameters:

(1) $$\eta_{t}=\frac{P_{r}}{P_{i}}=\eta_{r}\cdot \eta_{m}\cdot \left(1-{\left|S_{11}\right|}^{2}\right)$$

The first parameter is the radiation efficiency (*η_r_*), which depends on the physical properties of the ground plane. This is followed by the mismatch loss, which depends on the reflection coefficient of the antenna system (*S*_11_). Finally, the matching network efficiency (*η_m_*) represents the losses resulting from the relationship between the component losses (*Q_m_*) and the antenna’s quality factor (*Q_a_*). A simple equation for *η_m_* is obtained if all quality factors of the components of the matching networks are the same as *Q_m_* [18,19,20]:

(2) $$\eta_{m}=\frac{1}{1+\frac{Q_{a}}{Q_{m}}}$$

It is crucial to highlight that losses of the matching network do not depend on the *Q_m_* of the components but on the ratio of *Q_m_* over the *Q_a_*. For electrically large ground planes (length > λ/π), *Q_a_* is lower, so the efficiency of the matching can be high with high-quality factor components. However, when the ground plane is electrically small (<λ/π), *Q_a_* increases [9]. For example, if *Q_a_* = *Q_m_*, a matching network can bring an insertion loss of 3 dB. Thus, the mission of the slotted ground plane is lowering *Q_a_*, so losses of the matching network can be reduced compared to the no-slot case, as demonstrated in the next section.

Increasing the number of components can enhance antenna matching but also reduce the total efficiency due to higher component losses. Therefore, a balance must be struck between improved matching and efficiency losses.

The antenna quality factor can be obtained by the impedance data as [20,21]:

(3) $$Q_{a}(\omega )=\frac{\omega }{2R(\omega )}\sqrt{{\left[\frac{dR(\omega )}{d\omega }\right]}^{2}+{\left[\frac{dX(\omega )}{d\omega }+\left|\frac{X(\omega )}{\omega }\right|\right]}^{2}}$$

where *R*(*ω*) and *X*(*ω*) represent the real and imaginary components of the antenna system’s input impedance and *ω* is the angular frequency. The quality factor is inversely proportional to the bandwidth for a constant Standing Wave Ratio (*SWR*), as given by Equation (4).

(4) $$BW=\frac{f_{2}-f_{1}}{f_{0}}=\frac{SWR-1}{Q_{a}\cdot \sqrt{SWR}}$$

Note that Equations (3) and (4) allow us to estimate the bandwidth of an antenna system without the need for a matching network. There are some limitations when the impedance presents small impedance loops where the derivative diverges, and the estimation is not accurate. There are other alternatives called bandwidth potential, where the bandwidth of an antenna system is computed by adding a simple matching network of two components and then computing the bandwidth across frequency. This approach is more engineering and general since it avoids the limitations of the previous methods. This bandwidth potential is used as a metric next.

A high *Q_a_* value results in a narrowband antenna [19]. This narrowband behaviour makes the antenna more sensitive to component losses, which affects the mismatch loss and degrades the antenna’s reflection coefficient. As *Q_a_* increases, the total efficiency decreases due to poor impedance matching and greater matching losses. So, to enhance *η_t_*, a possible way is to reduce *Q_a_*, which can be achieved by increasing the bandwidth by manipulating the ground plane as proposed in this paper.

However, the efficiency of an antenna is inherently limited by the physical properties of its structure. No matter how much an antenna is tuned or adjusted, its efficiency cannot exceed certain physical limits. One way to evaluate the antenna’s performance is to calculate the first characteristic mode, also known as the fundamental mode [22,23,24]. The modal significance (MS) is an important metric that measures how effectively a particular mode can be excited. MS values range from 0 to 1, with a value of 1 meaning that the mode can be excited to its maximum amplitude, indicating peak performance for that mode [17]. The excitation of the fundamental mode occurs at 0.4 λ for a rectangular ground plane [9]. Therefore, it is crucial that the length of the ground plane is equal to 0.4 λ of the frequency of interest to achieve optimal mode excitation, resulting in improved bandwidth and efficiency. The next section puts this theory into practice.

## 3. Analysis

This section examines the impact of integrating slotted ground planes with antenna boosters on the total efficiency of wireless devices through a series of simulations and measurements.

Simulations were carried out to assess bandwidth potential, total efficiency, and modal significance for three different PCBs, each designed to cover the frequency band (824–960 MHz), representing typical wireless device dimensions [25]. Radiation efficiency, *S*_11_ parameter, and modal significance were calculated in CST Studio Suite [26], and these parameters (*η_r_* and *S*_11_) were managed in Optenni Lab software 6.1 [27] to process bandwidth potential and total efficiency *η_t_*. Once reference results were obtained, three different slot configurations were analysed for each PCB to evaluate their performance contributions.

Subsequently, measurements were performed on a slotted configuration applied to a 60 mm × 60 mm ground plane operating in the 824 MHz–960 MHz band, incorporating a battery on the PCB and assessing its impact on total efficiency.

Throughout the study, the antenna booster was modelled as a conductive parallelepiped with dimensions of 12 mm × 3 mm × 2.4 mm (width × depth × height).

### 3.1. Simulated Reference PCB

**Figure 1:**
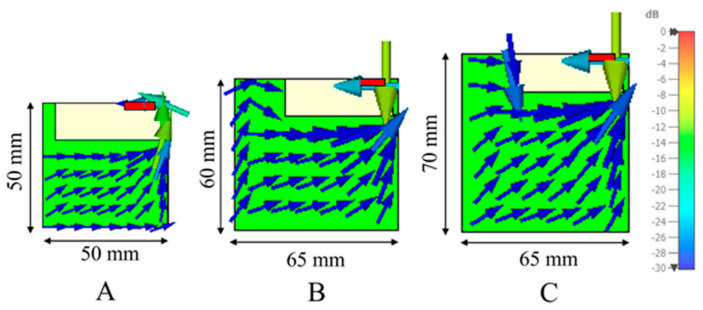
Three PCB sizes on a 1 mm thick FR4 substrate (ε_r_ = 4.15, tanδ = 0.02): 50 mm × 50 mm (PCB (**A**)), 60 mm × 65 mm (PCB (**B**)), and 70 mm × 65 mm (PCB (**C**)). Each PCB has a 15 mm × 45 mm clearance area and a 12 mm × 3 mm × 2.4 mm antenna booster placed at the top right corner. Current distributions are shown at 900 MHz.

This study evaluates the integration of an antenna booster into three PCB sizes: 50 mm × 50 mm (PCB A), 60 mm × 65 mm (PCB B), and 70 mm × 65 mm (PCB C). Each PCB includes a 15 mm × 45 mm clearance area (Figure 1), with corresponding ground plane dimensions of 35 mm × 50 mm for PCB A, 45 mm × 65 mm for PCB B, and 55 mm × 65 mm for PCB C. It is worth noting that a ground plane length of 50 mm corresponds to only 0.15 λ at 900 MHz, resulting in a small ground plane (length < λ/π).

Surface currents are illustrated to show that in the smallest PCB, the currents are predominantly excited along the longer side (the horizontal side). When the ground plane configuration changes from rectangular to square, both vertical and horizontal currents are excited (degenerate modes), as expected, since both the horizontal and vertical modes can be excited with the same intensity, resulting in a sort of diagonal current distribution.

As mentioned in Section 2, one way to improve the antenna’s total efficiency is to reduce the quality factor *Q_a_*, which effectively increases the bandwidth potential. Therefore, bandwidth potential is calculated first, as it is a quicker and simpler process. This calculation relies only on the *S*_11_ parameter, while the total efficiency depends on the radiation efficiency, *S*_11_, and the choice of components.

**Figure 2:**
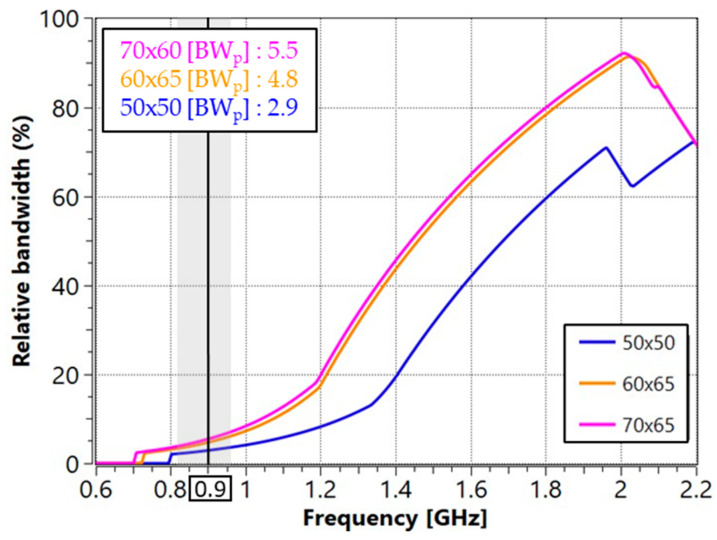
Bandwidth potential obtained with Optenni Lab at SWR ≤ 3. The grey area represents the 824–960 MHz frequency region.

Figure 2 shows the bandwidth potential evaluated using Optenni for a reference level of *SWR* ≤ 3 (*S*_11_ ≤ −6 dB) across the three PCB sizes. The results indicate that the bandwidth potential does not exceed 6% in the 824 MHz–960 MHz frequency band, with PCB A exhibiting 2.8%, PCB B achieving 4.6%, and PCB C reaching the highest value of 5.4%.

The average BW_p_ results suggest that larger PCBs are more likely to achieve higher efficiencies due to their greater bandwidth. However, it is important to note that the observed values are relatively low, especially compared to the requirements of, for example, cellular bands, which demand a BW_p_ of 15.1% at the centre frequency of 900 MHz to cover the entire 824 MHz–960 MHz frequency range.

Before evaluating total efficiency, an analysis will determine the most effective number of components for the matching network, aiming to balance improved antenna matching and minimal losses that could impact antenna efficiency. The matching network is obtained using Optenni Lab software, which provides a tool for designing the optimal MN based on specific requirements through matching network synthesis. This tool allows the specification of optimisation targets, which in our case are to achieve the maximum total efficiency in the 824 MHz to 960 MHz frequency band with a topology of 2/4/6 components. The matching network uses surface-mount device (SMD) components in 0402 and 0603 packages, including commercial inductors and capacitors with high *Q_m_* within the target frequency band [28]. For inductance values above 10 nH, SMD0603 are selected to ensure higher *Q_m_*. The following tables present the matching networks and associated parameters for each configuration (Table 2, Table 3 and Table 4).

The component analysis results (Table 5) demonstrate that increasing the number of components from two to four slightly improves total efficiency. For instance, PCB A shows a 3.1% increase, PCB B improves by 1.9%, and PCB C remains unchanged. However, adding more than four components provides limited benefits: PCB A shows minimal additional improvement, PCB B remains unchanged, and PCB C even decreases in efficiency. These findings suggest that using four components optimises performance across PCB sizes by maximising efficiency, minimising complexity, and avoiding unnecessary losses. However, from a practical perspective, two components in this case are enough.

**Figure 3:**
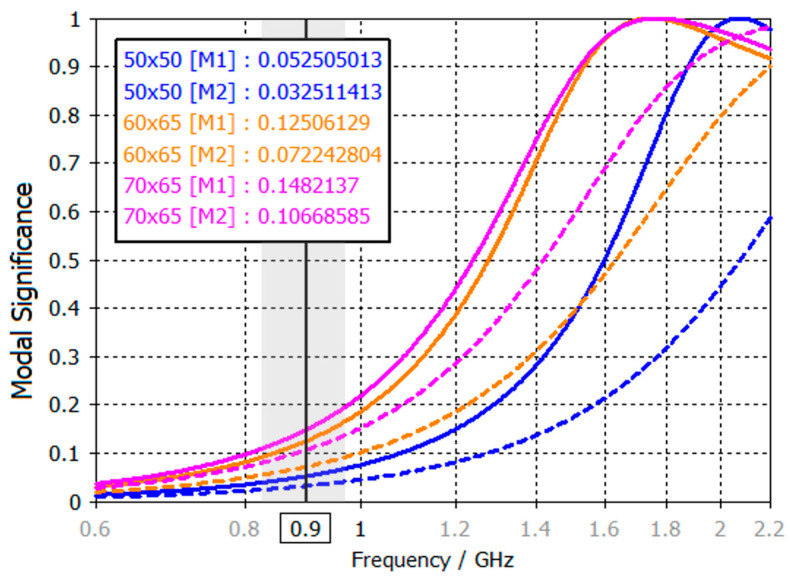
Modal significance of the 1st (M1) and 2nd modes (M2) for different PCB sizes. The grey area represents the 824–960 MHz frequency region.

In terms of PCB sizes, these results are consistent, as larger PCBs benefit from higher efficiency due to their physical properties. However, the efficiency results do not exceed 45% in the best case. As mentioned earlier, efficiency has certain limitations imposed by the physics of the ground plane. This phenomenon can be explained through modal significance (Figure 3), which evaluates the effectiveness of fundamental mode excitation across PCB sizes [22].

Figure 3 shows the modal significance of the fundamental mode, first mode, and second mode over different PCB sizes. When MS reaches a value of 1, the mode is fully excited. In this case, the fundamental mode reaches full excitation in the 1.7 GHz to 2 GHz frequency range. For PCBs between 50 mm and 70 mm, this corresponds to a physical length of approximately 0.4 λ, which explains the high BW_p_ values observed in this frequency range (Figure 2). However, in the frequency range of interest for cellular applications, the MS remains below 0.2 for all PCB sizes. This low MS suggests that the fundamental mode cannot be effectively excited in this range, which is to be expected since PCB sizes of 50 mm to 70 mm correspond to only 0.15 λ–0.2 λ. This fact inherently limits antenna performance as peak excitation typically occurs around 0.4 λ. These results highlight the limitations of small ground planes in resonating at lower frequencies.

Another detail to consider is the excitation behaviour of the second mode. For PCBs A and B, the second mode reaches half the value of the fundamental mode, while for PCB C, this value is slightly lower. As shown in Figure 1, this results in current distributions both horizontally (first mode, as it is the largest current path) and vertically (second mode), as both modes are similarly excited.

Given these challenges, it is essential to increase the electrical length of the ground plane to excite the fundamental mode at lower frequencies effectively. As detailed in the following section details, this is achieved by enlarging the ground plane with slots, which allows the fundamental mode to resonate within the 824 MHz–960 MHz band, ultimately improving performance.

### 3.2. Slotted Ground Plane

One way to increase the electrical length of the ground plane is to introduce a slot. This approach allows a longer current path without increasing the physical size of the PCB.

**Figure 4:**
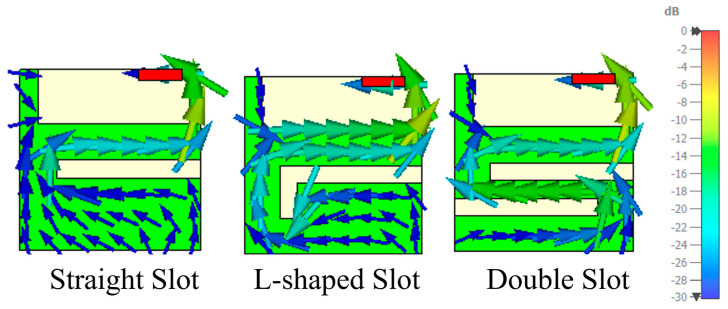
Three slot configurations: straight slot, L-shaped slot, and double slot. Currents are simulated with the same dynamic range and normalised to the same maximum. Current distribution is shown at 900 MHz.

The slot side position and dimensions (distance between the feeding point and the slot and slot length) are adjusted to achieve the maximum bandwidth potential (Appendix A), keeping the slot width constant at 5 mm. The insertion of a straight slot in the PCB has caused the currents to follow a longer path, thus increasing the bandwidth. Upon analysing potential locations for the slot, it is determined that the right edge of the PCB offers the most significant bandwidth potential (being the BW_p_, the criterion selected to select the most effective configuration). Therefore, the slot is placed along the right edge (Figure 4).

**Figure 5:**
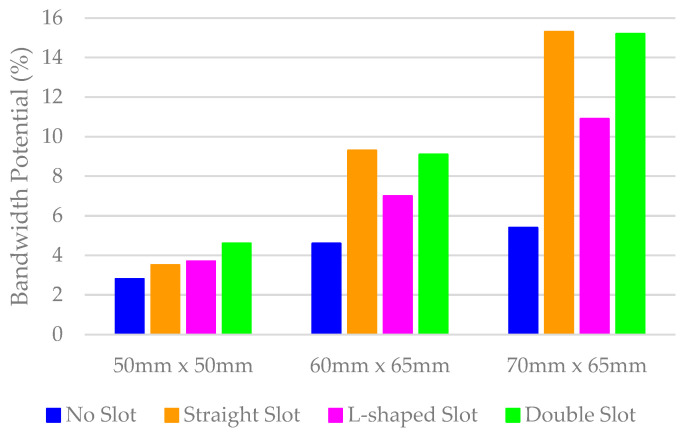
Simulated bandwidth potential (824 MHz–960 MHz) for four PCB models across three different PCB sizes obtained with Optenni Lab software.

The straight-slot configuration is not the only solution. Other configurations, such as the L-shape and double slot, can be used to increase the current path, further improving the bandwidth potential (Figure 5). The analysis of the bandwidth potential shows two different behaviours. The addition of the slot significantly increases bandwidth, with an improvement of up to 2 times for PCB B, increasing from 4.6% to 9.3%, and 2.8 times for PCB C, increasing from 5.4% to 15.3%. However, for PCB A, the improvement is more modest, rising from 2.8% with no slot ground plane to 4.6% with the double slot configuration.

**Figure 6:**
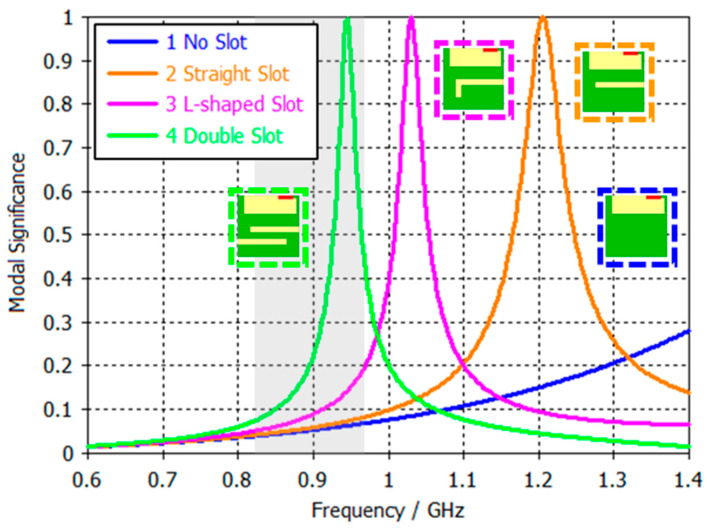
Modal significance of the fundamental mode for 50 mm × 50 mm PCB without and with slots. The grey area represents the 824–960 MHz frequency region.

Despite the modifications introduced in PCB A (50 mm × 50 mm), its smaller size limits its ability to be effectively excited within the target frequency band, as shown in Figure 6. Even if the current path of the PCB is increased, the fundamental mode in the 824 MHz–960 MHz band cannot be exited for both straight and L-shaped slot configurations, resulting in lower efficiency compared to other PCBs.

**Figure 7:**
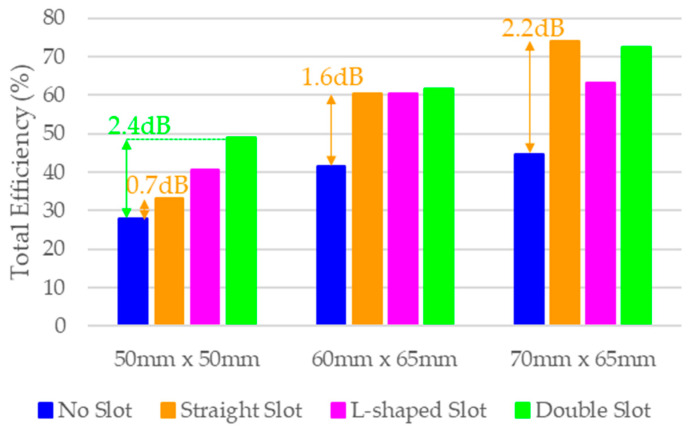
Simulated average total efficiency (824 MHz–960 MHz) for four PCB models across three different PCB sizes.

As discussed in the previous section, the efficiency of each model is calculated using a matching network with four components that maximises total efficiency in each case within the 824 MHz to 960 MHz band (Figure 7). The straight slot significantly improves efficiency across all PCB sizes, increasing from 44.7% to 74.1% in PCB C, equivalent to 2.2 dB. The L-shaped slot shows similar performance but is more effective in the smallest PCB (28.1% to 40.7%), which is expected as smaller PCBs require greater electrical length extension to operate within the same frequency band. The double slot configuration achieves the highest total efficiency (28.1% to 49%, 2.4 dB more) in PCB A, suggesting that multiple slots further increase the electrical length.

**Figure 8:**
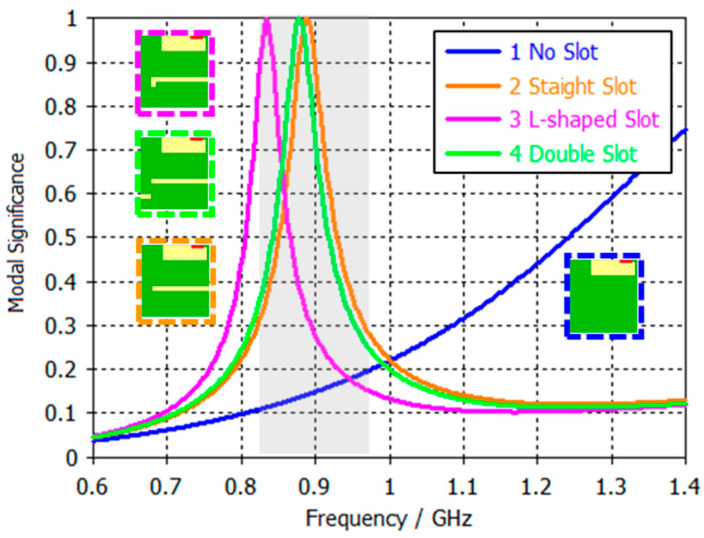
Modal significance of the fundamental mode for 70 mm × 65 mm PCB variations. The grey area represents the 824–960 MHz frequency region.

It is worth noting the significant differences between the straight slot and the L-shaped slot in PCBs B and C (Figure 8). While the straight slot offers a wider bandwidth, the L-shaped slot is better matched to the band of interest, implying a lower quality factor. This lower *Q_a_* reduces its sensitivity to *Q_m_*, minimising *η_m_* and resulting in a slight improvement in efficiency (Table 6). Table 6 reflects this fact. First, we assume the same quality factor *Q_m_* for the matching network. Since *Q_a_* is lower for the slotted ground plane, the matching network efficiency *η_m_* is higher. Thus, the product *η_r_·η_m_* is 0.9 dB higher than the case without the slot. Now, when we request both systems to operate at 824–960 MHz, the case without a slot fails to achieve a good S11 across the band since it only reaches 5.4% (Figure 5), whereas the slotted case reaches 15.3%. As a result, there is more mismatch loss for the non-slotted ground plane, which, in, the end, considering both effects, mismatching plus the *η_r_·η_m_*, results in 2.2 dB more efficiency (Figure 7).

After evaluating several solutions to improve antenna efficiency, the L-shaped slot emerges as the preferred choice for PCBs B and C due to its superior performance in improving efficiency over the targeted frequency range. This design approach significantly enhances the antenna system performance.

However, the straight slot is advantageous from a practical manufacturing point of view. Its design accommodates a larger available area on the PCB, which facilitates the integration of additional components. This increased area is beneficial in real-world scenarios where space constraints for additional electronic elements are critical. Consequently, while the L-shaped slot offers greater efficiency, the straight slot may be more suitable for practical PCB fabrication, balancing performance with component integration requirements.

**Figure 9:**
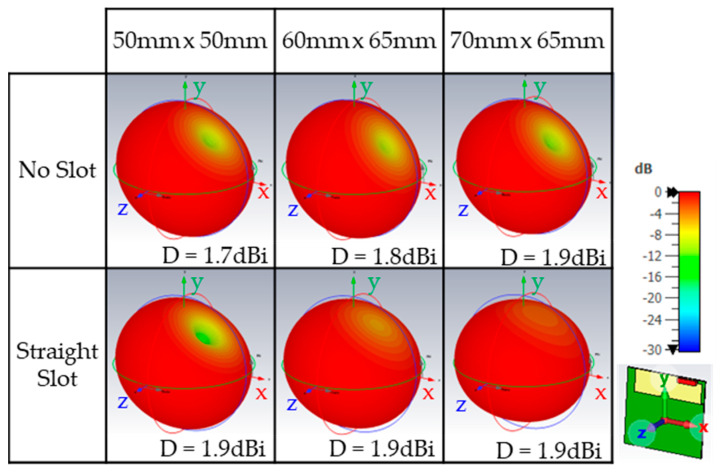
Simulated 3D radiation pattern showing the directivity at 900 MHz.

To further analyse the effect of the proposed slot configurations, Figure 9 shows the 3D radiation patterns for each configuration. These patterns show a quasi-isotropic radiation pattern with a directivity of approximately 2 dBi, which is advantageous for IoT devices. In IoT communications, the direction of the incoming signal and the orientation of the device are typically random, making this radiation pattern highly suitable for such applications.

**Figure 10:**
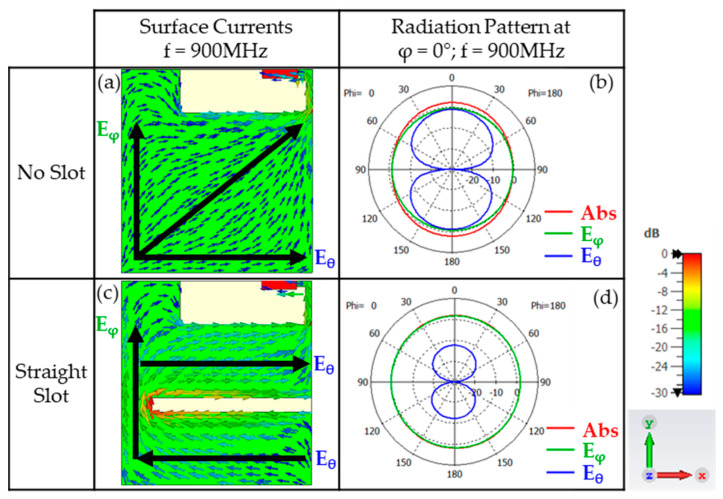
Simulated surface currents and radiation pattern at φ = 0° for an antenna at 900 MHz with a 70 mm × 65 mm PCB, comparing the no-slot and straight-slot configurations. For current distributions, the black arrows have intentionally added to highlight the main current contribution. For (**a**), the net current is mainly tilted 45° resulting in the radiation pattern at (**b**). For (**c**), the main current distribution is vertical (y-axis) resulting in a omnidirectional radiation pattern represented by E_ϕ_ (**d**); E_θ_ is due to the contribution of the horizontal currents resulting in a E_θ_ field with less amplitude than E_ϕ_ since the horizontal currents are out-of-phase and electrically close.

Examining the radiation pattern at θ = 0° for the no-slot configuration (Figure 10b), both the theta and phi components exhibit the same magnitude. With an axial ratio of 30 dB in this direction, the polarisation is linear along the X-Y plane, as observed for the surface currents (Figure 10a), where the current flows obliquely.

For the straight-slot configuration at θ = 0° (Figure 10d), the phi component dominates over the theta component (axial ratio = 16.6 dB), resulting in a dominant polarisation along the Y-axis. This can also be seen in the surface currents (Figure 10c), where the currents along the Y-axis are stronger, while those along the X-axis tend to cancel each other out.

Therefore, the addition of the slot does not alter the linear polarisation of the antenna.

## 4. Experimental Validation

Having seen the bandwidth and efficiency benefits when including slots in the ground plane, a prototype was designed with a small ground plane. Its performance was improved by adding a slot according to the lessons learned in the previous section.

**Figure 11:**
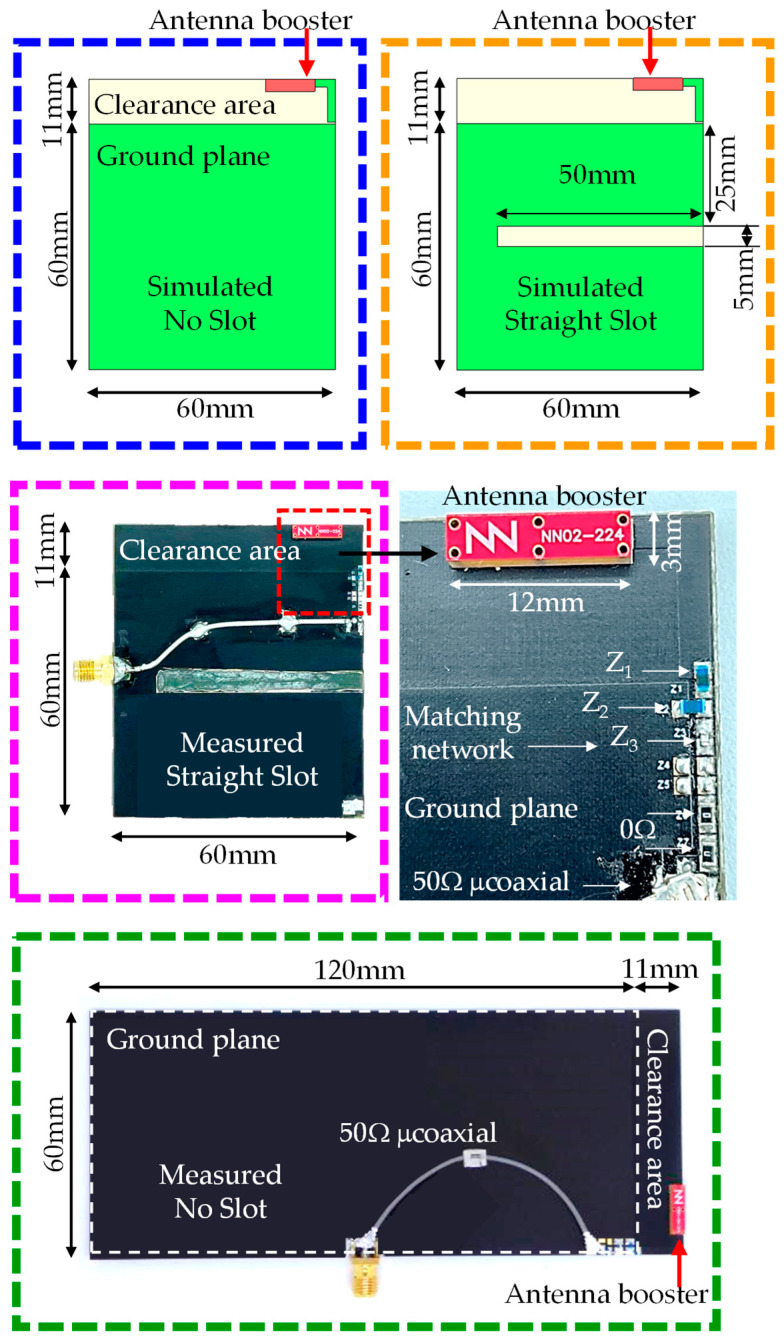
Simulated and measured PCBs, including a 12 mm × 3 mm × 2.4 mm (height) antenna booster. Both simulated cases and prototypes include a thin 1 mm thick substrate, ε_r_ = 4.15, tanδ = 0.02. For the prototypes, the matching network is included in the top right corner of the ground plane, including a 50 Ω micro coaxial line ending with an SMA connector for testing *S*_11_ and efficiency. Colours for each case are linked with the data shown in Figure 12.

For illustrative purposes, two ground planes were implemented and measured: a 60 mm × 60 mm ground plane (0.18 λ at 900 MHz) and a 120 mm × 60 mm ground plane (0.36 λ at 900 MHz) (Figure 11). The 60 mm × 60 mm configuration included an 11 mm × 60 mm clearance area and a 50 mm length × 5 mm width slot positioned 25 mm from the feeding point, focusing on the frequency band between 824 MHz and 960 MHz. A simple matching network consisting of an L series, an L shunt, and a C series was used (Table 7). The matching network can be identified in Figure 11, where Z_1_ = 22 nH is the component closest to the antenna booster. The 120 mm × 60 mm configuration also included an 11 mm × 60 mm clearance area and employed a matching network with an L series and an L shunt (Table 8). In this case, the first series component was closest to the antenna booster. A matching network with finite high Q components was synthesised for the simulated ground planes using the Optenni Lab software.

**Figure 12:**
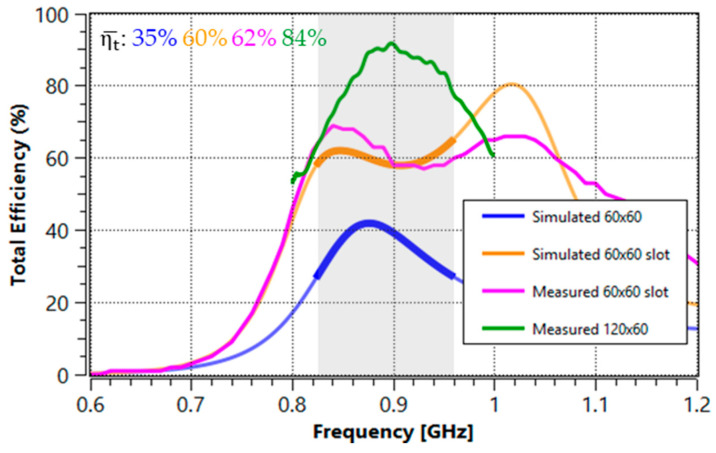
Total efficiency results obtained from the PCBs in Figure 11. The average total efficiency from 824 MHz to 960 MHz is included in the numbers. The grey area represents the 824-960 MHz frequency region. Labels in the legend indicate the size of the ground plane.

The simulation of a straight slot on the 60 mm × 60 mm ground plane (orange) shows a notable improvement in total efficiency compared to the ground plane without a slot (blue), achieving efficiencies of 60% and 35%, respectively (Figure 12). This improvement represents a significant increase of 2.3 dB over the frequency range of interest. Such an increase in efficiency is attributed to the inclusion of the slot, demonstrating that modifying the ground plane geometry has a substantial positive effect on antenna performance.

For the measurement results, an anechoic chamber (MVG Starlab 18 [29]) was used to obtain the total efficiency using 3D pattern integration. When comparing the results of the simulated slotted ground plane (orange) with the measured version (pink), a remarkable agreement is observed: 60% in simulation and 62% in measurement. This similarity is due to the consideration of factors such as dielectric losses and losses in the matching network components, although the influence of measurement cables, the SMA connector, and the pads was not included in the simulation. The correlation between simulation and measurement validates the efficiency improvement achieved with the slot in the ground plane and confirms that the results can be effectively implemented in practice.

In addition, measurements were taken on a larger 120 mm × 60 mm ground plane (green), with a total efficiency of 84%. This comparison shows that the inclusion of the slot not only significantly improves the performance of the smaller ground plane but also achieves results closer to those of the larger ground plane, with a difference of only 1.3 dB.

### Battery Impact

Batteries are needed to power the electronics, such as the transceiver of the wireless device. Therefore, to analyse the practical feasibility of the solution, a battery was integrated into the PCB. As the FR4 substrate was 1 mm thick, the battery was separated from the ground plane by 1 mm. The placement of the battery was carefully considered to minimise interference with the performance of the antenna system, ensuring that the compact design maintained maximum efficiency while providing the necessary power for additional components.

**Figure 13:**
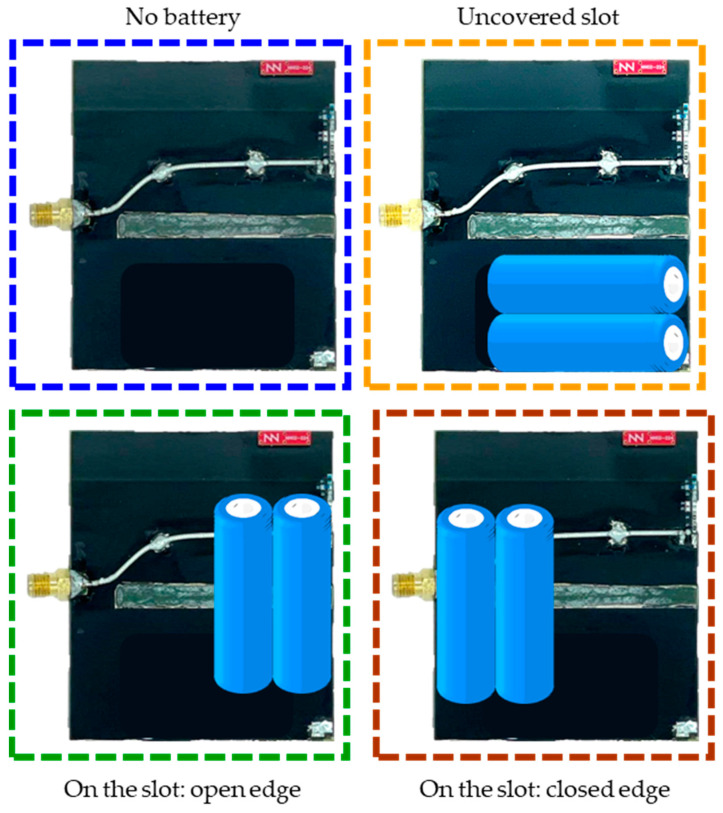
Battery (AA type) placement on the PCB.

The first configuration serves as a reference with no battery present. The not-covering slot configuration positions the battery horizontally under the slot along the open edge. In contrast, the other two configurations position the battery perpendicular to the slot: one along the open edge and the other along the closed edge, where the battery aligns with the edge of the slot (Figure 13).

**Figure 14:**
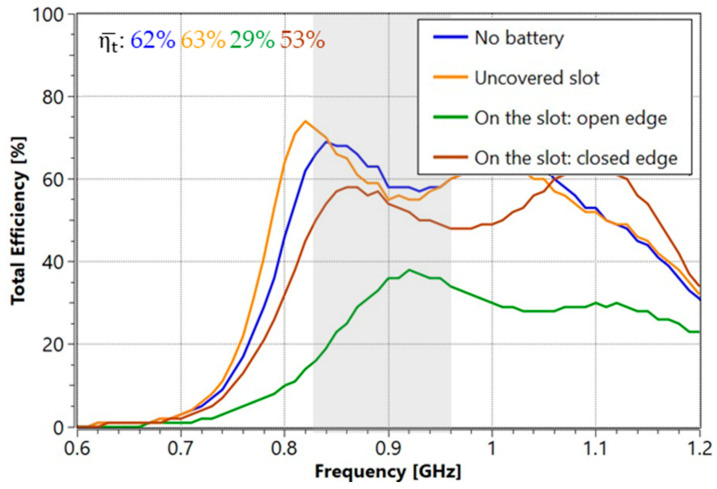
Measured total efficiency results when the battery is included on the ground plane. The average total efficiency is from 824 MHz to 960 MHz. The grey area represents the 824–960 MHz frequency region.

Figure 14 shows the measured total efficiency results for different battery placement configurations. The no-battery configuration serves as a reference and achieved a baseline efficiency of 62%, averaged from 824 MHz to 960 MHz. When the battery was not covering the slot, the efficiency remained the same as the reference, indicating minimal interference with antenna performance.

However, the efficiency dropped significantly in the vertical configuration, reaching 29% at the open edge. This reduction suggests notable interference with the antenna system’s performance, likely due to the battery’s proximity to the open edge of the slot. Similarly, the closed-edge configuration results in an efficiency of 53%, representing a considerable decrease compared to the reference. From a practical standpoint, if the battery does not interfere with the slot aperture, the efficiency is maintained compared to the reference case without the batteries.

## 5. Conclusions

The proposed solution demonstrates that including slots in the ground plane is an effective strategy for increasing the electrical length of small wireless devices (length < λ/π), enabling them to achieve performance closer to that of larger devices. Incorporating a slot allows the fundamental mode of these smaller devices to be excited within the 824–960 MHz band, resulting in a substantial 2 dB improvement in efficiency over this frequency range.

## Figures and Tables

**Table 1:** Comparative table between relevant works.

Reference	Ground Plane Size (mm × mm)	Frequency (MHz)	Electric Length (λ)
[11]	90 × 40	850–900, 1800–1900	0.25
[12]	107 × 40	900	0.32
[15]	90 × 35	900, 1900	0.27
This work	50 × 50–70 × 65	824–960	0.15–0.2

**Table 2:** Two-component matching network.

Matching Network	Symbol	Value	*Q_m_*
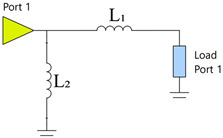	L_1_	24 nH	87
	L_2_	12 nH	86

**Table 3:** Four-component matching network.

Matching Network	Symbol	Value	*Q_m_*
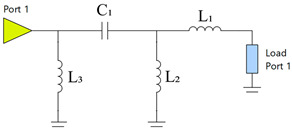	L_1_	27 nH	85
	L_2_	2.3 nH	74
	L_3_	2.7 nH	64
	C_1_	6.8 pF	253

**Table 4:** Six-component matching network.

Matching Network	Symbol	Value	*Q_m_*
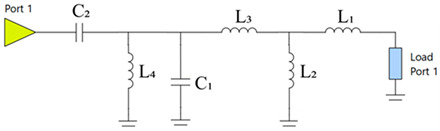	L_1_	27 nH	85
	L_2_	4.1 nH	90
	L_3_	3.3 nH	73
	L_4_	7.9 nH	72
	C_1_	6.4 pF	258
	C_2_	2.7 pF	380

**Table 5:** Average total efficiency for different PCB sizes with 2, 4, and 6 component matching network within the 824 MHz–960 MHz frequency range.

Number of Components	*η_t_* A	*η_t_* B	*η_t_* C
2	25.1%	39.8%	44.7%
4	28.2%	41.7%	44.7%
6	28.8%	41.7%	42.6%

**Table 6:** Comparison of antenna parameters with and without slot at 900 MHz.

Antenna Parameter	70 × 65 mm^2^ PCB No Slot	70 × 65 mm^2^ PCB Straight Slot
*η_r_*, radiation efficiency	92.6%	95.7%
*Q_m_*	60	60
*Q_a_* (based on Equations (3) and (4))	21.3	7.5
*η_m_* (based on Equation (2))	73.8%	88.8%
*η_r·_η_m_*	68.3	84.9

**Table 7:** Matching network for the slotted 60 mm × 60 mm ground plane.

Component	Value	Size	*Q_m_*
L series	22 nH	0603	87
L shunt	8.7 nH	0402	75
C series	2 pF	0402	446

**Table 8:** Matching network for the slotted 120 mm × 60 mm ground plane.

Component	Value	Size	*Q_m_*
L series	30 nH	0603	88
L shunt	20 nH	0603	88

**Figure A1:**
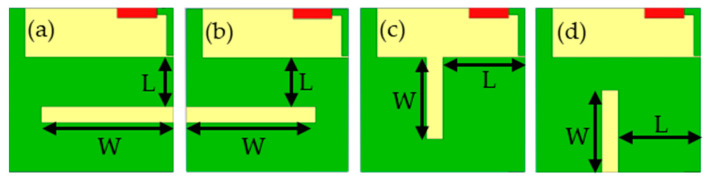
PCB A: Slot Placement Analysis. (**a**) Right (**b**) Left (**c**) Top (**d**) Bottom.

**Figure A2:**
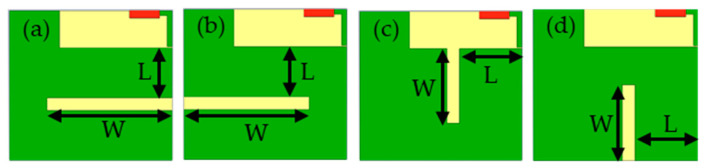
PCB B: Slot Placement Analysis. (**a**) Right (**b**) Left (**c**) Top (**d**) Bottom.

**Figure A3:**
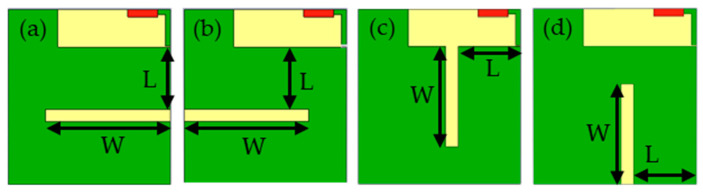
PCB C: Slot Placement Analysis. (**a**) Right (**b**) Left (**c**) Top (**d**) Bottom.

## Data Availability

The original contributions presented in the study are included in the article. Further inquiries can be directed to the corresponding author.

## Abbreviations

The following abbreviations are used in this manuscript:

BW	Bandwidth
IoT	Internet of Things
MDPI	Multidisciplinary Digital Publishing Institute
mm	Millimetres
MN	Matching Network
MS	Modal significance
NB	Narrowband
PCB	Printed circuit board
PIFA	Planar Inverted F Antenna
SMD	Surface-mount devices
SWR	Standing Wave Ratio

## Appendix A

### Appendix A.1. First Round

A preliminary round determines which side (right, left, top, or bottom) is most suitable for slot placement. This is carried out by sweeping through L (the distance between the feeding point and the slot) and W (the slot length) (Figure A1, Figure A2 and Figure A3) for each possible position, calculating the bandwidth potential at each point. The slot width remains fixed at 5 mm in all cases. Finally, the slot is placed on the side with the highest BW_p_.

**Table A1:** PCB A—Right side: Average bandwidth potential (in %) from 824 MHz to 960 MHz.

W ↓–L →	10 mm	15 mm	20 mm
10 mm	2.9	2.9	2.9
25 mm	3.0	3.0	3.0
40 mm	3.5	3.4	3.2

**Table A2:** PCB A—Left side: Average bandwidth potential (in %) from 824 MHz to 960 MHz.

W ↓–L →	10 mm	15 mm	20 mm
10 mm	2.8	2.8	2.8
25 mm	2.9	2.9	2.9
40 mm	2.8	3.0	3.0

**Table A3:** PCB A—Top side: Average bandwidth potential (in %) from 824 MHz to 960 MHz.

W ↓–L →	10 mm	25 mm	40 mm
10 mm	3.0	2.9	2.9
15 mm	3.2	3.1	3
20 mm	3.3	3.2	3.1

**Table A4:** PCB A—Bottom side: Average bandwidth potential (in %) from 824 MHz to 960 MHz.

W ↓–L →	10 mm	25 mm	40 mm
10 mm	2.9	2.8	2.8
15 mm	2.9	2.9	2.8
20 mm	3.0	3.0	2.9

**Table A5:** PCB B—Right side: Average bandwidth potential (in %) from 824 MHz to 960 MHz.

W ↓–L →	10 mm	20 mm	30 mm
10 mm	4.7	4.7	4.7
30 mm	5.1	5.0	4.9
50 mm	6.5	7.1	5.9

**Table A6:** PCB B—Left side: Average bandwidth potential (in %) from 824 MHz to 960 MHz.

W ↓–L →	10 mm	20 mm	30 mm
10 mm	4.6	4.6	4.6
30 mm	4.7	4.7	4.7
50 mm	5.4	5.7	5.1

**Table A7:** PCB B—Top side: Average bandwidth potential (in %) from 824 MHz to 960 MHz.

W ↓–L →	10 mm	25 mm	40 mm
10 mm	4.9	4.9	4.8
20 mm	5.0	5.2	4.9
30 mm	6.3	6.8	6.3

**Table A8:** PCB B—Bottom side: Average bandwidth potential (in %) from 824 MHz to 960 MHz.

W ↓–L →	10 mm	25 mm	40 mm
10 mm	4.7	4.7	4.7
20 mm	4.8	4.8	4.8
30 mm	5	5.2	2

**Table A9:** PCB C—Right side: Average bandwidth potential (in %) from 824 MHz to 960 MHz.

W ↓–L →	10 mm	25 mm	40 mm
10 mm	5.5	5.4	5.4
30 mm	6.2	6.1	5.7
50 mm	11.3	11.9	8.0

**Table A10:** PCB C—Left side: Average bandwidth potential (in %) from 824 MHz to 960 MHz.

W ↓–L →	10 mm	25 mm	40 mm
10 mm	5.4	5.4	5.4
30 mm	5.5	5.5	5.4
50 mm	7.4	8.4	6.5

**Table A11:** PCB C—Top side: Average bandwidth potential (in %) from 824 MHz to 960 MHz.

W ↓–L →	10 mm	25 mm	40 mm
10 mm	5.5	5.4	5.2
25 mm	6.4	6.5	6.2
40 mm	9.0	11.3	10.9

**Table A12:** PCB C—Bottom side: Average bandwidth potential (in %) from 824 MHz to 960 MHz.

W ↓–L →	10 mm	25 mm	40 mm
10 mm	5.4	5.4	5.4
25 mm	5.5	5.6	5.5
40 mm	6.2	6.7	6.3

In all three PCBs (PCB A, B and C) the optimal positions are the sides in contact with the feeding point (right and top side). In this case, the right side is selected as it performs slightly better than the top side, although the latter remains a valid alternative.

### Appendix A.2. Second Round

A second round is conducted to refine the slot position on the right side and determine its length in more detail.

**Table A13:** PCB A—Right side: Average bandwidth potential (in %) from 824 MHz to 960 MHz.

W ↓–L →	5 mm	10 mm	15 mm
30 mm	3.2	3.1	3.1
35 mm	3.3	3.3	3.2
40 mm	3.5	3.5	3.4

**Table A14:** PCB B—Right side: Average bandwidth potential (in %) from 824 MHz to 960 MHz.

W ↓–L →	15 mm	20 mm	25 mm
45 mm	6.0	5.9	5.7
50 mm	7.3	7.1	6.5
55 mm	7.5	9.3	9.0

**Table A15:** PCB C—Right side: Average bandwidth potential (in %) from 824 MHz to 960 MHz.

W ↓–L →	15 mm	20 mm	25 mm
45 mm	8.6	8.5	8.2
50 mm	12.8	12.6	11.9
55 mm	13.7	14.9	15.3

**Table A16:** Position (L and W) of the best slot placement for all three PCBs.

	PCB A	PCB B	PCB C
L (mm)	10	20	25
W (mm)	40	55	55
